# Unintentional Drug Overdose Mortality in Years of Life Lost Among Adolescents and Young People in the US From 2015 to 2019

**DOI:** 10.1001/jamapediatrics.2021.6032

**Published:** 2022-01-31

**Authors:** O. Trent Hall, Candice Trimble, Stephanie Garcia, Parker Entrup, Megan Deaner, Julie Teater

**Affiliations:** 1 Ohio State University Wexner Medical Center Talbot Hall, Department of Psychiatry and Behavioral Health, Columbus; 2 Riverside, California; 3 College of Medicine, The Ohio State University, Columbus

## Abstract

This cross-sectional study assesses the mortality among adolescents and young people in the US from 2015 to 2019 in years of life lost from unintentional drug overdose.

Unintentional drug overdose has become a grave and sustained public health burden in the US.^1^ The US Centers for Disease Control and Prevention (CDC) defines unintentional drug overdose as occurring “…when no harm is intended.”^2^^(p1) ^and inclusive of “…overdoses resulting from drug misuse, drug abuse, and taking too much of a drug for medical reasons.”^2^^(p1) ^Adult decedents have been the focus of most overdose mortality reports, despite the fact that adolescents (aged 10-19 years) and young people (aged 10-24 years) are increasingly dying of unintentional drug overdose.^3^ This troubling trend requires further study, given that adolescents and young people are deprived of many more years of work, community life, and family life than are older individuals dying of unintentional drug overdose.

To our knowledge, no prior study has assessed unintentional drug overdose mortality among adolescents and young people in years of life lost (YLL). YLL is an epidemiologic descriptor that gives weight to deaths among the young.^4^ YLL analysis has the potential to provide important context to the overdose crisis by better representing what is meant to society by the loss of adolescents and young people to unintentional drug overdose. The present work aimed to fill this important gap in the literature by calculating unintentional drug overdose YLL in this vulnerable population.

## Methods

This cross-sectional retrospective study involved summary-level death records from January 1, 2015, to December 31, 2019, obtained from the CDC’s Wide-Ranging Online Data for Epidemiologic Research (CDC WONDER) mortality file.^5^ YLL were calculated as standard life expectancy minus age at death. Male and female life expectancy at each individual age was determined from the 2017 Social Security Administration Period Life Table. Information on race and ethnicity was not gathered to protect the privacy of the individuals in the database. Decedents were identified by the *International Statistical Classification of Diseases and Related Health Problems, Tenth Revision *codes X40-X44. The Ohio State University Wexner Medical Center institutional review board approved this study and granted a waiver of patient consent owing to the use of deidentified patient data. This study followed the Strengthening the Reporting of Observational Studies in Epidemiology (STROBE) reporting guidelines.

## Results

A total of 3296 adolescents (aged 10-19 years) died of unintentional drug overdose in the US between 2015 and 2019 (Figure). The mean (SD) age at death for adolescent unintentional drug overdose decedents was 15.1 (2.7) years. Male adolescents outnumbered female adolescents in incident deaths (2267 [68.8%] vs 1029 [31.2%]) and YLL (133 023.64 vs 65 548.28). Annual total YLL due to unintentional drug overdose was stably elevated with a mean (SD) 39 714.38 (2689.63) annual YLL (Table). Adolescents experienced a total of 187 077.92 YLL during the study period.

**Figure.**
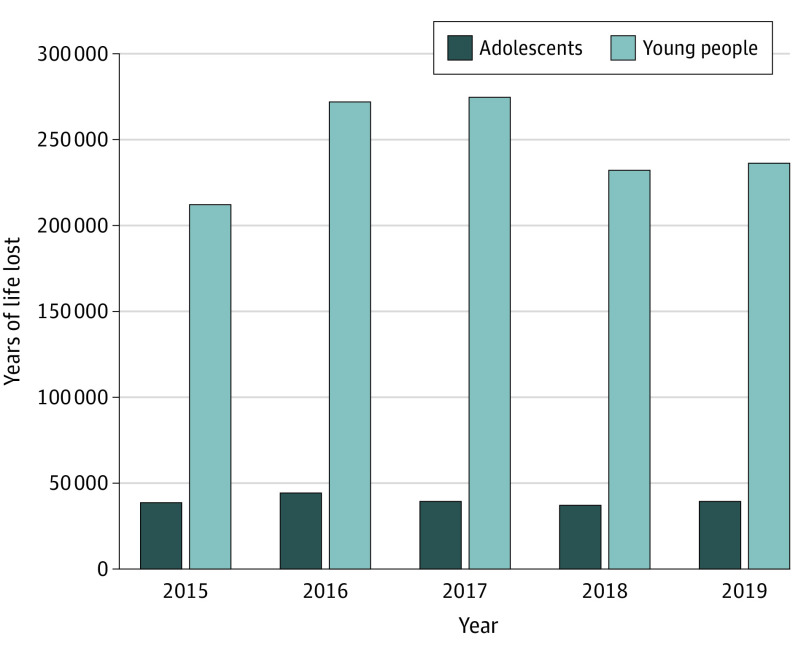
Years of Life Lost to Unintentional Drug Overdose Among Adolescents and Young People From 2015 to 2019

**Table.** Annual Mortality Due to Unintentional Drug Overdose Among Adolescents and Young People, 2015-2019

Year	Boys/men		Girls/women		Overall	
	Deaths, No.	YLL	Deaths, No.	YLL	Deaths, No.	YLL
**Adolescents**						
2015	436	25 578.86	202	12 885.74	638	38 464.60
2016	514	30 159.22	220	14 028.38	734	44 187.60
2017	455	26 662.98	201	12 815.28	656	39 478.26
2018	407	23 910.59	207	13 117.02	614	37 027.61
2019	455	26 711.99	199	12 701.86	654	27 919.85
Total	2267	133 023.64	1029	65 548.28	3296	187 077.92
**Young people**						
2015	2694	148 984.31	1050	63 219.62	3744	212 203.93
2016	3574	197 270.24	1239	74 608.05	4813	271 878.29
2017	3465	190 957.17	1398	83 744.29	4863	274 701.46
2018	2871	158 439.46	1227	73 727.14	4098	232 166.60
2019	3000	165 925.24	1171	70 348.06	4171	236 273.30
Total	15 604	861 576.42	6085	365 647.16	21 689	1 227 223.58

Abbreviation: YLL, years of life lost.

A total of 21 689 young people (aged 10-24 years) died of unintentional drug overdose (Figure). The mean (SD) age at death for young people who died of unintentional drug overdose was 17.6 (4.1) years. Male young people outnumbered female young people in incident deaths (15 604 [71.9%] vs 6085 [28.1%]) and YLL (861 576.42 vs 365 647.16) (Table). Young people experienced a total of 1 227 223.58 YLL during the 5-year period of study.

## Discussion

Over the 5-year period of this cross-sectional study, adolescents experienced nearly 200 000 YLL, and young people amassed greater than 1.25 million YLL. Male adolescents and young people accounted for substantially greater unintentional drug overdose mortality (YLL and incident deaths) than female adolescents and young people. Although limited by death records potentially undercounting overdoses and a cross-sectional design insensitive to temporal relations between risk factors and deaths, our findings represent an unacceptable preventable mortality burden for adolescents and young people in the US. Prior research has identified polysubstance use, psychiatric comorbidity, and unstable housing as relevant risk factors for unintentional drug overdose in this age cohort.^6^ Our findings suggest that further resources are needed to mitigate these factors. The present study should inform future mortality reviews among adolescents and young people, as well as ecologic interventions involving family, school, and community, in unintentional drug overdose prevention and substance use treatment.
